# Standardized Scalp Massage Results in Increased Hair Thickness by Inducing Stretching Forces to Dermal Papilla Cells in the Subcutaneous Tissue

**Published:** 2016-01-25

**Authors:** Taro Koyama, Kazuhiro Kobayashi, Takanori Hama, Kasumi Murakami, Rei Ogawa

**Affiliations:** ^a^Men's Health Clinic Tokyo, Tokyo, Japan; ^b^Department of Plastic, Reconstructive and Aesthetic Surgery, Nippon Medical School, Tokyo, Japan; ^c^ANGFA Co, Ltd, Tokyo, Japan

**Keywords:** hair, dermal papilla cells, scalp massage, mechanobiology, stretching force

## Abstract

**Objective:** In this study, we evaluated the effect of scalp massage on hair in Japanese males and the effect of stretching forces on human dermal papilla cells in vitro. **Methods:** Nine healthy men received 4 minutes of standardized scalp massage per day for 24 weeks using a scalp massage device. Total hair number, hair thickness, and hair growth rate were evaluated. The mechanical effect of scalp massage on subcutaneous tissue was analyzed using a finite element method. To evaluate the effect of mechanical forces, human dermal papilla cells were cultured using a 72-hour stretching cycle. Gene expression change was analyzed using DNA microarray analyses. In addition, expression of hair cycle-related genes including IL6, NOGGIN, BMP4, and SMAD4 were evaluated using real-time reverse transcription-polymerase chain reaction. **Results:** Standardized scalp massage resulted in increased hair thickness 24 weeks after initiation of massage (0.085 ± 0.003 mm vs 0.092 ± 0.001 mm). Finite element method showed that scalp massage caused *z*-direction displacement and von Mises stress on subcutaneous tissue. In vitro, DNA microarray showed gene expression change significantly compared with nonstretching human dermal papilla cells. A total of 2655 genes were upregulated and 2823 genes were downregulated. Real-time reverse transcription-polymerase chain reaction demonstrated increased expression of hair cycle–related genes such as NOGGIN, BMP4, SMAD4, and IL6ST and decrease in hair loss–related genes such as IL6. **Conclusions:** Stretching forces result in changes in gene expression in human dermal papilla cells. Standardized scalp massage is a way to transmit mechanical stress to human dermal papilla cells in subcutaneous tissue. Hair thickness was shown to increase with standardized scalp massage.

Aging results in a decrease in the total number and thickness of hair, termed androgenetic alopecia (AGA) in the male and female pattern hair loss (FPHL) in the female population.^1^ Both represent a major concern in appearance in the affected population. Finasteride, an oral 5-α reductase inhibitor, is an effective drug to prevent progression of AGA.^2^ Minoxidil, an ATP-sensitive potassium channel opener, enhances hair growth in both AGA and FPHL.^3^^,^^4^ While these medications are effective, some patients are not completely satisfied with the results. Moreover, many patients are hesitant to start medication or consider their hair loss as not severe enough to start medication. Therefore, many people turn toward supplements, hair care products, and scalp massage with the expectancy to increase hair growth. Scalp massage, in addition to having a relaxing effect, results in increased blood flow and skin softening.^5^ Therefore, patients have the notion that it can also increase hair growth rates. However, the effect of scalp massage on hair growth has not been evaluated in sound clinical trials and its effect remains unclear.^6^

Organs, tissues, and cells are constantly exposed to mechanical forces and subsequently react to them. For example, blood vessels are subject to shear stress of blood flow, bones receive pressure due to weight bearing, cartilage is exposed to hydrostatic pressure by weight bearing, and hypertrophic scars develop with increased tension to the wound.^7^ We hypothesize that scalp massage is a way to deliver mechanical forces to the scalp including epidermis, dermis, skin appendages, blood vessels, and nerves. In this study, we evaluated the effect of scalp massage on hair number, thickness, and growth rate. We also used finite element method (FEM) to analyze the mechanical effect of scalp massage on the subcutaneous tissue and the dermal papilla cells in the hair follicle. Finally, we evaluated the effect of mechanical forces on human dermal papilla cells (hDPCs), which play an important role in hair growth due to interaction with hair matrix cells. Mechanical stress to the scalp was applied via stretching forces by scalp massage.

## MATERIALS AND METHODS

### Effect of scalp massage on human hair in 9 healthy men

**Figure 1:**
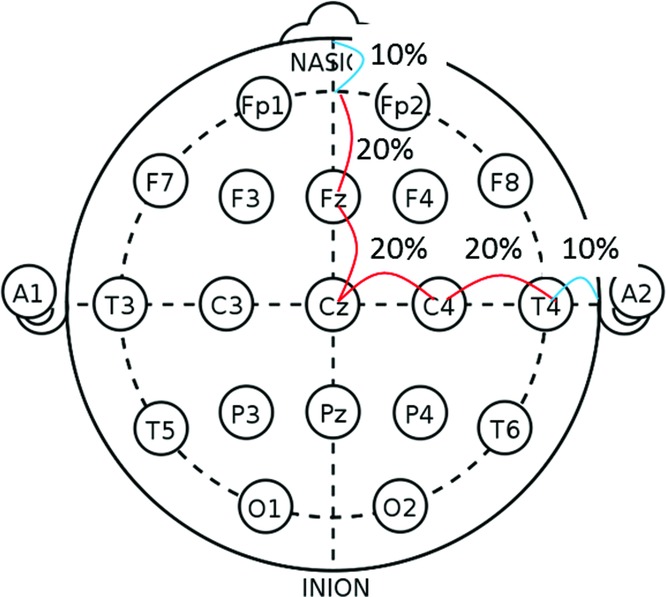
C3 and C4 determined by International 10-20 system for electroencephalography were applied for the massage area and the control area.

Nine healthy Japanese men aged 25 to 46 (mean ± SD = 34.8 ± 8) years participated in the study. The study subjects showed no obvious hair loss. In the study, they received scalp massage using a scalp massage device, Panasonic EH-HM75 (Panasonic, Osaka, Japan), on one side of their temporal scalp every day for 4 minutes at 170 rpm. International 10-20 system for electroencephalography was used to define the area for scalp massage (Fig 1). The symmetrical position on the contralateral side served as a control without any massage treatment. The massage and control sides were chosen randomly for each subject. Total hair number, thickness, and growth speed were measured at 0, 4, 12, and 24 weeks following initiation of massage. The massage area and the control area were clipped prior to measurement of hair count and hair thickness. Three days after clipping, hair length was measured to evaluate the hair growth rate. A folliscope (LeadM Corporation, Seoul, Korea) was used for measurement. Each parameter in the massage area and the control area was compared with the initial point. The tenets of the Declaration of Helsinki were followed, and the written informed consent was obtained from the volunteers.

### Effect of scalp massage on subcutaneous tissue using FEM

The geometry of the scalp massage device and scalp was reproduced to evaluate the mechanical effect of scalp massage to subcutaneous tissue by using KSWAD Ver 7.10 (Kubota System Inc, Osaka, Japan) and ADVENTURECluster Win Ver4.5 (Allied Engineering Corporation, Tokyo, Japan). The scalp was divided into 5 layers including epidermis, dermis, subcutaneous tissue, galea, and bone. We set mechanical property of thickness, Young modulus, and Poisson ratio in each layer from previous reports (Table 1). Composite component displacement of the skin surface, *z*-direction displacement of the subcutaneous tissue, and von Mises stress of the subcutaneous tissue were analyzed.

### Cell cultures

Human dermal papilla cells (ScienCell Research Laboratories, Carlsbad, CA) were thawed according to manufacturer's instructions and plated at a density of 2500 cells/cm^2^ in 10 mL of culture media comprising Dulbecco's Modified Eagle Medium supplemented with 10% fetal bovine serum (Thermo Fisher Scientific, Waltham, MA) and 1% Antibiotic-Antimycotic (Thermo Fisher Scientific, Waltham, MA). The cells were expanded at 37°C with 5% CO_2_ and 90% humidity until they reached 80% to 90% confluence. At passages 3, the hDPCs were seeded into a silicone chamber at a density of 1 × 10^5^ cells/chamber. The chamber measured 32 × 32 × 10 mm in dimensions and consisted of a 400-mm-thick outer wall and a 200-mm-thick membrane bottom.

### Application of mechanical stretch

To generate the stretch group, the hDPCs were allowed to attach for 24 hours to the chamber surface in 5 mL of culture medium. After changing the medium, the silicon chambers were attached to a stretching apparatus driven by a computer-controlled step motor (STB-140; Strex Ltd, Osaka, Japan). Continuous uniaxial sinusoidal stretch (10 cycles/min) was applied at 37°C, 5% CO_2_ for 72 hours. The stretching stimulation was set at 20% stretch. To generate the control group, the culture medium was changed and the silicon chambers were incubated as described earlier for 72 hours without stretching.

### DNA microarray analysis

RNA was isolated by using standard RNA extraction protocols (NucleoSpin_ RNAII), and its quality was checked using the Agilent 2100 Bioanalyzer platform (Agilent Technologies, Santa Clara, Calif) through gel imaging and with an electropherogram. Each total RNA sample (100 ng) was used for linear T7-based amplification. The resulting cRNA (complementary RNA) was measured by using a ND-1000 spectrophotometer (Thermo NanoDrop, Wilmington, Del). The control samples were labeled with Cy3, whereas the experimental samples were labeled with Cy5. Hybridization was performed using the Agilent Gene Expression Hybridization Kit (Agilent Technologies). The fluorescence signals generated by the hybridized Agilent oligo microarrays were detected using the Agilent DNA microarray scanner (Agilent Technologies). The data were extracted from images using the Agilent Feature Extraction Software.

### Gene expression evaluation using real-time reverse transcription-polymerase chain reaction

Real-time polymerase chain reaction (PCR) was performed using a standard TaqMan PCR kit protocol on an Applied Biosystems 7900HT Sequence Detection System (P/N: 4329002; Applied Biosystems, Carlsbad, CA). The 10 µL PCR solution included 0.67 µL of RT product, 1× TaqMan Universal PCR Master Mix (P/N: 4324018; Applied Biosystems), 0.2 µM TaqMan probe, 1.5 µM forward primer, and 0.7 µM reverse primer. The incubation reactions were performed in a 384-well plate at 95°C for 10 minutes, followed by 40 cycles of 95°C for 15 seconds and 60°C for 1 minute. All reactions were run in triplicate. The threshold cycle (CT) is defined as the fractional cycle number at which the fluorescence passes the fixed threshold. TaqMan CT values were converted into absolute copy numbers using a standard curve. GAPDH (glyceraldehyde-3-phosphate dehydrogenase) served as the internal control. The following primers were used: Homo sapiens bone morphogenetic protein 4 (BMP4), transcript variant 1, mRNA [NM_001202], Homo sapiens NOGGIN (NOG), mRNA [NM_005450], Homo sapiens SMAD family member 4 (SMAD4), mRNA [NM_005359], Homo sapiens interleukin 6 (interferon, beta 2) (IL6), mRNA [NM_000600], Homo sapiens interleukin 6 signal transducer (gp130, oncostatin M receptor) (IL6ST), transcript variant 1, mRNA [NM_002184]. Relative expression was calculated using the 2^−△△Ct^ method with a correction for different amplification efficiencies.

### Statistical analysis

A Man-Whitney *U* test was used for comparison of total hair counts, hair thickness, and hair growth rate between the scalp massage area and the control area. In experiments using microarray and real-time reverse transcription (RT)-PCR, statistical comparisons were carried out by means of analysis of variance. Results were considered significant when *P* value was less than .05.

## RESULTS

### Effect of scalp massage on human hair in 9 healthy men

**Figure 2:**
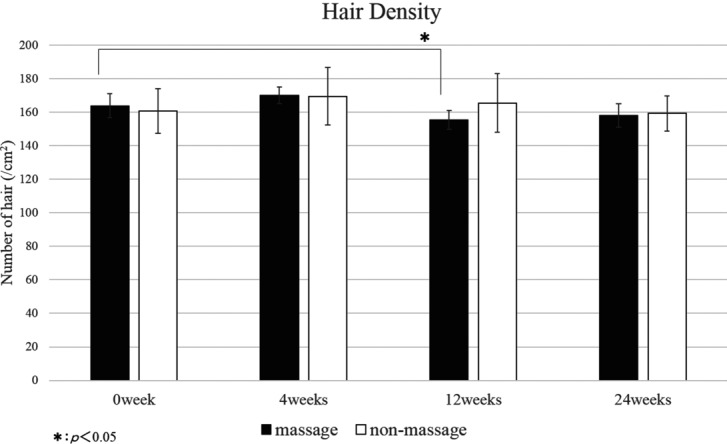
Change from baseline in hair density. Throughout the 24-week study period, hair density showed no significant difference between the massage area and the control area. Hair density in the massage area showed significant decrease at 12 weeks compared with baseline (163.889 ± 7.237/cm^2^ vs 155.500 ± 5.607/cm^2^). Hair density in the control area showed no significant change at any point compared with baseline.

**Figure 3:**
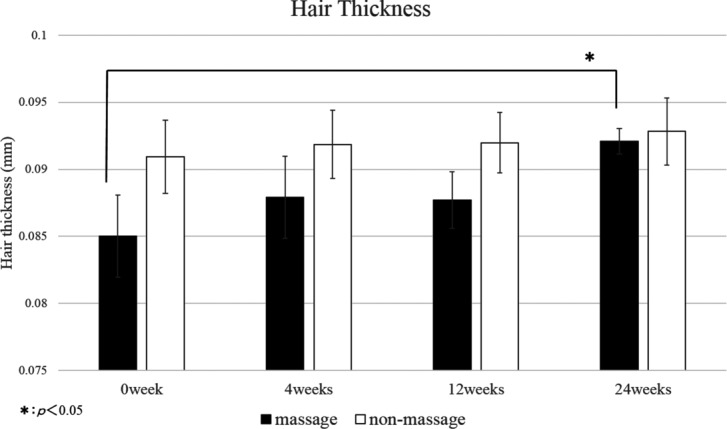
Change from baseline in hair thickness. Throughout the 24-week study period, hair thickness showed no significant difference between the massage area and the control area. Hair thickness in the massage area showed significant increase at 12 weeks compared with baseline (0.085 ± 0.003 mm vs 0.092 ± 0.001 mm). Hair thickness in the control area showed no significant change at any point compared with baseline.

**Figure 4:**
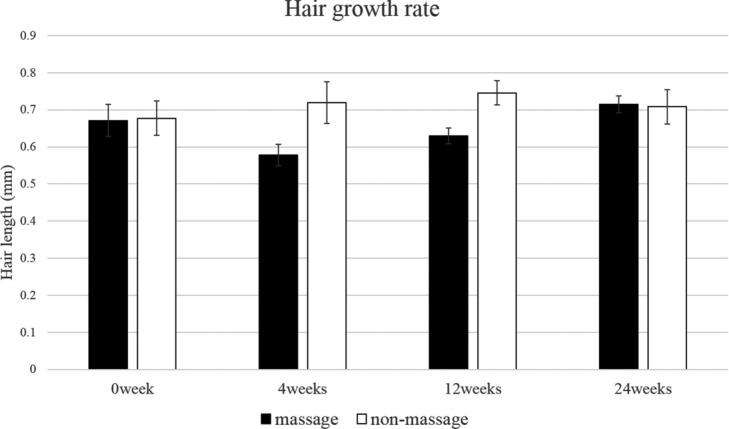
Change from baseline in hair growth rate. Throughout the 24-week study period, hair growth rate showed no significant difference between the massage area and the control area. In addition, hair growth rate both in the massage area and in the control area showed no significant change at any point compared with baseline.

The control area showed no significant change in hair count, hair thickness, and hair growth rate throughout the 24-week study period (Figs 2–4). The scalp massage area showed no significant change in the hair growth rate throughout the study period (Fig 4). However, the scalp massage area showed a significant increase in hair thickness at 24 weeks compared with the initiation point (0.085 ± 0.003 mm vs 0.092 ± 0.001 mm) and significant decrease in hair count at 12 weeks (163.889 ± 7.237/cm^2^ vs 155.500 ± 5.607/cm^2^) (Figs 2 and 3).

### Effect of scalp massage on subcutaneous tissue using FEM

**Figure 5:**
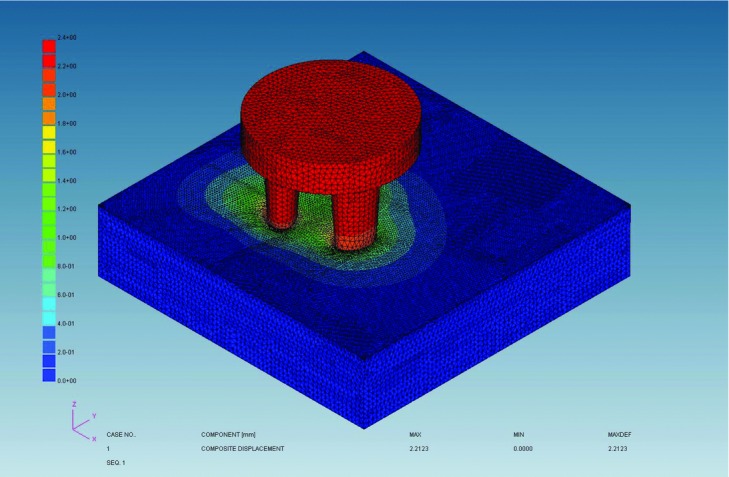
Image demonstrating horizontal skin surface (green) movement with the scalp massage device (red).

**Figure 6:**
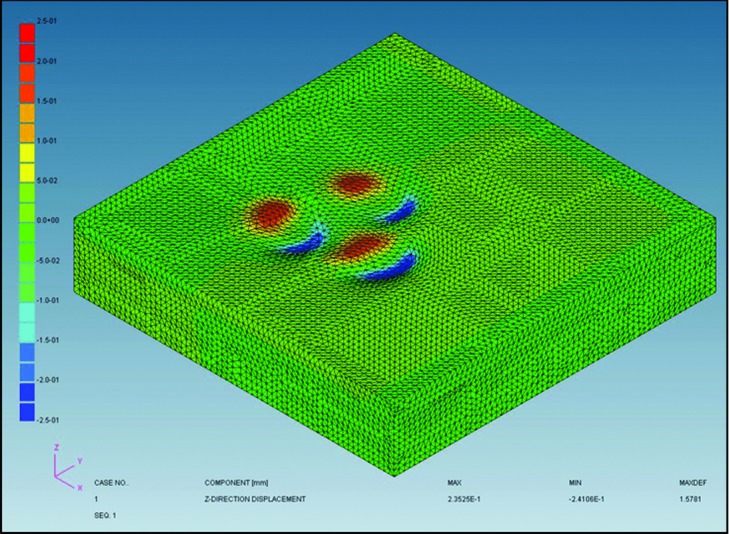
*z*-direction displacement occurred in subcutaneous tissue like a wave, whereas the scalp massage device shows movement of skin surface only in *x*-direction.

**Figure 7:**
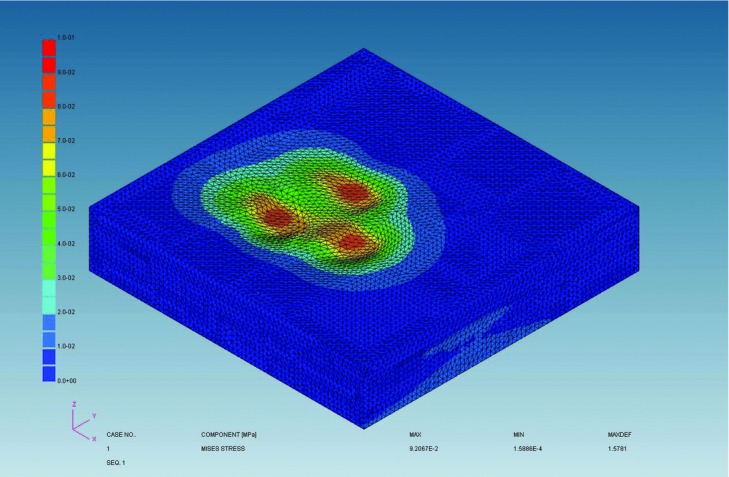
von Mises stress occurred in subcutaneous tissue. Stress is the highest in the middle of the wave.

Finite element method demonstrated that scalp massage resulted in horizontal movement of skin surface and *z*-direction displacement of the subcutaneous tissue (Figs 5 and 6); *z*-direction displacement shows a wave pattern. The maximum von Mises stress was shown in the center of the displaced area (Fig 7).

### DNA microarray analyses

Following 72 hours of stretching hDPCs, 2655 genes were upregulated and 2823 genes were downregulated more than 2.0-fold compared with genes in the control group (Table 2).

### Gene expression evaluation using real-time RT-PCR

**Figure 8:**
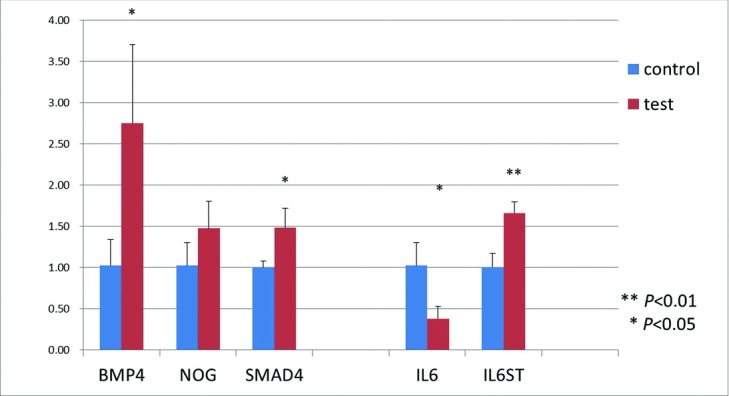
IL6 was downregulated in stretching hDPCs compared with control cells. BMP4, NOGGIN, SMAD4, and IL6ST were upregulated in stretching hDPCs compared with control cells. hDPCs indicates human dermal papilla cells.

Among the genes screened by microarray analysis, hair cycle–related genes including BMP4, NOGGIN, SMAD4, IL6, and IL6ST were tested using real-time RT-PCR. IL6 was downregulated in hDPCs after 72 hours of stretching compared with control cells, and BMP4, NOGGIN, SMAD4, and IL6ST were upregulated in hDPCs after 72 hours of stretching when compared with control cells (Fig 8).

## DISCUSSION

### Mechanobiology and mechanotherapy

At the cellular level, cells can change shape, and molecules are transferred into and out of the cell through the cell membrane. These processes are all driven by mechanical forces, which activate molecular cascades within the cells that alter gene expression and thereby play an important role in the life cycle of the cell. “Mechanobiology” is the study of those molecular cascades and cellular responses: key questions relate to how cells sense and respond to the mechanical forces of the physical microenvironment. A more comprehensive and improved understanding of mechanobiology may greatly facilitate the development of new therapies that control mechanical forces and thereby specifically induce desired molecular, cellular, tissue, and/or organ formation, changes, or repair. Recently, we denoted these therapies as “mechanotherapies.” While the term “mechanotherapy” was originally synonymous with physical therapy, massotherapy, and the rehabilitation of musculoskeletal systems, our new definition reflects the multidimensional medical possibilities of the field now. An example of potential mechanotherapies was recently proposed by our article,^8^ which suggested that various skin disorders, including keloids, can be treated by mechanobiological methods. Moreover, mechanobiology-mediated medicine could be used to analyze and/or treat many disorders/diseases, accelerate wound healing, reduce scarring during wound healing, and repair and regenerate injured/aged tissues and organs.^7^

At present, we are studying mechanobiology and mechanotherapy at both the basic research and clinical levels. Specifically, we are searching for methods to regenerate nail and hair and are conducting research in the medical fields of aesthetics and antiaging.^9^

### Mechanotherapy for hair: Effects of scalp massage

Hair number decreased in the massage area at 12 weeks after initiation of standardized scalp massage. Some telogen hair in the massage area might have fallen by scalp massage and decrease in the number of hair occurred temporarily. On the contrary, hair thickness increased significantly at 24 weeks after initiation of standardized scalp massage. Improvement in blood flow is one possible explanation for hair thickness improvement as previously reported^10^; however, this was not evaluated in the current study. In addition to an increase in blood flow, direct stimulation of mechanical force to dermal papilla cells would be another explanation for increased hair thickness. Using FEM, we demonstrated that *z*-direction displacement of subcutaneous tissue including dermal papilla cells was initiated by standardized scalp massage. We also demonstrated that mechanical stress such as von Mises stress was transmitted to the subcutaneous tissue layer. We did not perform tissue biopsies following scalp massage and therefore were not able to analyze any histological changes in hair follicles after scalp massage; however, we could show that stretching forces changed the gene expression in hDPCs in vitro. These genes included hair cycle–related genes such as IL6, IL6ST, BMP4, NOGGIN, and SMAD4.^11^^-^13 Therefore, scalp massage could have the potential to influence hDPCs via stretching the scalp skin. Mechanical stress has been reported to influence various signaling pathways in many different cell groups. For example, stretching forces have been shown to stimulate Wnt signaling in dermal fibroblasts as well as in bone.^14^^,^^15^ In hair follicles, Wnt signaling accelerated the anagen phase of the hair cycle.^16^^,^^17^ In addition, ATP-sensitive potassium channels in the atrium have been shown to be mechanosensitive.^18^ Minoxidil used as hair growth medication is an ATP-sensitive potassium channel opener that works on ATP-sensitive potassium channels in dermal papilla cells.^19^ If optimal stretching forces in hair follicles are applied for an appropriate time period to stimulate hair growth genes selectively, mechanical forces could be useful in hair tissue engineering. Moreover, scalp massage as a form of mechanical stimulation of the scalp may become a natural, easy, and economical way to stimulate hair growth rates. Further research is required to confirm these possibilities.

## CONCLUSION

We demonstrated changes in gene expression in hDPCs following application of stretching forces in vitro. By FEM analysis, scalp massage has been shown to induce mechanical stress on hDPCs. Hair thickness increased following standardized scalp massage in healthy Japanese men.

## Figures and Tables

**Table 1:** Mechanical property of scalp for finite element method analysis

	Mechanical property		
	Thickness, mm	Young modulus, MPa	Poisson ratio
Epidermis	0.0614	0.85	0.4
Dermis	1.4643	0.85	0.4
Subcutaneous tissue	1.9289	0.182	0.4
Galea	1.5569	0.85	0.4
Skull	6.1	17,000	0.25

**Table 2:** Up- and downregulated genes related to hair cycle in human hair dermal papilla cells in response to 72 -hour stretching determined by microarray hybridization

Upregulated genes	
WNT1	6.65
FGF9	6.23
VEGF-D	5.38
FGF12	5.14
BMP4	4.29
WISP1 (WNT inducible signal. path. protein)	4.26
HBEGF (heparin-binding EGF-like GF)	3.36
PGF (placental growth factor)	3.36
PDGF-D	2.66
TNFaSF11b	2.52
TNFaSF10a	2.57
TNFaSF11a	2.59
WNT11	2.42
TGFbR3	2.29
VEGF-C	2.04
Downregulated genes	
IL6	−2.69
FGF7	−2.39
PDGF-A	−2.34
TNFaIP2	−2.19
TNFaIP8	−2.11
